# Autoimmune Gastritis and *Helicobacter pylori* Infection: Molecular Mechanisms of Relationship

**DOI:** 10.3390/ijms26167737

**Published:** 2025-08-11

**Authors:** Dmitry S. Bordin, Maria A. Livzan, Sergei I. Mozgovoi, Olga V. Gaus

**Affiliations:** 1 A. S. Loginov Moscow Clinical Scientific Center, Department of Pathology of the Pancreas, Bile Ducts and Upper Digestive Tract, 111123 Moscow, Russia; 2 Department of Propaedeutic of Internal Diseases and Gastroenterology, Russian University of Medicine, 127473 Moscow, Russia; 3 Department of Outpatient Therapy and Family Medicine, Tver State Medical University, 170100 Tver, Russia; 4 Department of Internal Medicine and Gastroenterology, Omsk Sate Medical University, 644099 Omsk, Russia; mlivzan@yandex.ru (M.A.L.); gaus_olga@bk.ru (O.V.G.); 5 Department of Pathological Anatomy, Omsk Sate Medical University, 644099 Omsk, Russia; simozgovoy@yandex.com

**Keywords:** autoimmune gastritis, *Helicobacter pylori*, molecular mimicry, gastric adenocarcinoma, neuroendocrine neoplasia, *H. pylori*-associated gastritis, eradication therapy

## Abstract

*Helicobacter pylori* (*H. pylori*) infection and autoimmune inflammation of the gastric mucosa are recognized as the leading etiological factors of chronic atrophic gastritis. The mechanisms of atrophy formation and progression with the risk of gastric cancer development are heterogeneous, which requires a deeper study of the molecular mechanisms of relationship, peculiarities of the course of autoimmune gastritis both in combination with *H. pylori* and after eradication, as well as without *H. pylori* infection (naïve AIG). This article presents the specific molecular and cellular patterns in the formation of these related conditions.

## 1. Introduction

*Helicobacter pylori* (*H. pylori*) infection and autoimmune inflammation of the gastric mucosa are recognized as leading etiological factors in the development of atrophic gastritis [1]. It should be noted that the mechanisms of inflammation development and their procarcinogenic potential differ, making it critically important to compare the pathogenetic factors both under conditions of isolated action of each etiological factor and their combined effects.

A comprehensive understanding of the molecular mechanisms driving autoimmune inflammation, particularly in the context of *H. pylori* coexisting infection, paves the way for optimizing preventive strategies against AIG in *H. pylori*-associated gastritis and developing innovative programs to monitor inflammatory changes in the gastric mucosa.

**Figure 1:**
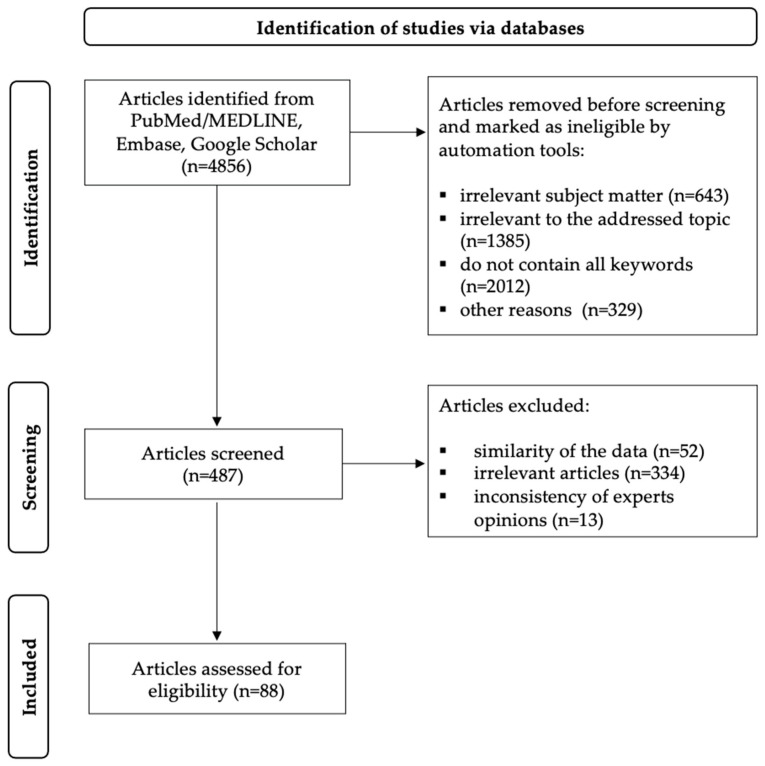
PRISMA flow diagram for article selection.

This publication aims to systematize available data on autoimmune gastritis (AIG), including the characteristics of atrophy development and associated carcinogenesis risks—both in isolated autoimmune inflammation and in cases of persistent or prior *H. pylori* infection. A systematic search of articles was conducted in the PubMed/MEDLINE, Embase, and Google Scholar databases. The search used combinations of the following keywords based on Medical Subject Headings (MeSHs), including “autoimmune gastritis”, “*H. pylori*-associated gastritis”, “post-*H. pylori* eradication gastritis”, and “*H. pylori* molecular mimicry.” The article selection criteria for this review were developed in accordance with international guidelines outlined in the PRISMA standard (Preferred Reporting Items for Systematic Reviews and Meta-Analyses) [2]. Articles relevant to the review topic were included in the analysis, published in English in authoritative and high-impact journals up to May 2025. The selection comprised clinical observations, original studies, systematic reviews, and meta-analyses that provided clear, methodologically sound descriptions to ensure data reproducibility and validity. Exclusion criteria comprised studies published in languages other than English, abstracts, editorials, letters to the editor, preprints, and conference proceedings. All authors independently screened publications for eligibility by reviewing titles, abstracts, and full texts. Data extraction was likewise performed individually by each author. For disputed studies, a consensus-based approach was implemented—sources were excluded unless unanimous agreement among all coauthors was reached (any single dissenting vote resulted in exclusion). After applying the selection criteria, a total of 88 articles were included in this review. Figure 1 presents the search algorithm utilizing the PRISMA flow diagram.

Autoimmune gastritis is a chronic autoimmune disease affecting the body and fundus of the stomach, characterized by an immune response directed against parietal cells and intrinsic factors. According to data from several authors, the prevalence of AIG in the population ranges from 1% to 8%, occurring more frequently in women compared to men, in a 3:1 ratio [1]. Autoimmune inflammation of the gastric mucosa develops through the interaction of genetic and environmental factors.

The genetic risk factors for AIG remain insufficiently studied. Initial research focused on identifying associations between AIG risk and haplotypes of the human leukocyte antigen (HLA) system, whose main effect is antigen presentation to immune cells [3]. For example, Ungar B. et al. described an increased frequency of HLA-B8, HLA-B18, and HLA-Bw*15 haplotypes in patients with AIG and pernicious anemia [4]. Another study revealed an increased frequency of HLA-DR2 or HLA-DR4 and HLA-DR4 or HLA-DR5 in patients with pernicious anemia [5]. The heterogeneity of HLA haplotypes in different clinical subgroups may reflect the genetic heterogeneity of pernicious anemia and possibly AIG [3].

There are two studies in the literature regarding predisposition to AIG depending on HLA gene variations [6,7]. In an Italian population among 89 AIG patients, the prevalence of HLA-DRB1*03 and HLA-DRB1*04 was higher compared to healthy individuals (28.1% vs. 15.9%, *p* = 0.01, and 25.8% vs. 14.4%, *p* = 0.01, respectively) [6]. Furthermore, HLA-DRB1*03 and HLA-DRB1*04 were associated with the presence of intestinal metaplasia (*p* < 0.01). In a Finnish population among 12 AIG patients with severe atrophy of the gastric body mucosa, HLA-DRB1*04 and DQB1*03 were significantly more frequently detected (83%) than in the general population (58% vs. 28%, *p* = 0.045, and 83% vs. 51%, *p* = 0.034, respectively) [7]. These HLA haplotypes are also frequently associated with other autoimmune conditions, thereby supporting a common HLA-dependent pathway for autoimmune pathology development [3].

Among the genes studied as potentially associated with AIG are ATP4A and ATP4B, which are involved in the synthesis of the α and β transporting subunits of the specific transmembrane enzyme hydrogen–potassium adenosine triphosphatase (H^+^/K^+^-ATPase), and thus in maintaining hydrochloric acid secretion [8]; the autoimmune regulator (AIRE) gene, which encodes a thymic transcription factor playing a key role in the elimination of autoreactive T cells [9]; the BACH2 gene, which encodes one of two transcription factors from the Bach family (BTB domain and Cap‘n’Collar homolog) involved in controlling immune responses through transcriptional repression of target genes [10]; and the SLC4A2, SLC26A7, and SLC26A9 genes, which encode members A2, A7, and A9 of the solute carrier families 4 and 26, playing a key role in modulating acid–base balance and H^+^/K^+^ exchange in parietal cells [11].

A meta-analysis of a genome-wide association study involving 2166 patients with pernicious anemia and 659,516 individuals from population biobanks identified the following genes associated with pernicious anemia: Protein Tyrosine Phosphatase, Non-Receptor Type 22 (PTPN22), Polyribonucleotide Nucleotidyltransferase 1 (PNPT1), Major Histocompatibility Complex Class II (MHC II) DQ beta 1 haplotype (HLA-DQB1), Interleukin-2 Receptor Alpha Subunit (IL2RA), and AIRE [12].

Beyond established genetic contributors to gastric mucosal inflammation, significant research focus has shifted toward immune response-related genes, particularly those governing Toll-like receptor (TLR) expression [13]. As critical components of innate immunity, TLRs serve as primary sensors for bacterial pathogen-associated molecular patterns (PAMPs) and damage-associated molecular patterns (DAMPs) released from compromised host cells. The human TLR family comprises ten members, several of which demonstrate constitutive and inducible expression in gastric epithelium. While most TLRs promote inflammatory cascades and proliferative responses during infection and malignant transformation [14], TLR9 exhibits context-dependent duality—either exacerbating or suppressing inflammation based on microenvironmental cues [15]. Mechanistically, TLR activation initiates downstream signaling cascades, including nuclear factor kappa B (NF-κB) and inflammasome pathways, ultimately upregulating genes encoding pro-inflammatory mediators (cytokines, chemokines), antigen-presenting molecules, and co-stimulatory factors essential for adaptive immunity [16]. Notably, TLRs orchestrate host defense against *H. pylori* while potentially modulating carcinogenic processes [17]. Multiple *H. pylori* components—lipopolysaccharide (LPS), peptidoglycan, and microbial nucleic acids—function as PAMPs detectable by specific TLRs. TLR2/TLR4, for instance, serve as primary receptors for bacterial peptidoglycans and LPSs [18], with their engagement triggering an elevated secretion of interleukin 8 (IL-8), interleukin 1β (IL-1β) and tumor necrosis factor α (TNFα)—central mediators of inflammatory initiation and perpetuation [19]. Emerging evidence suggests TLR-driven inflammation dynamically shapes T-helper cell (Th) responses (particularly Th1/Th17 differentiation), which are pivotal for anti-*H. pylori* immunity [20]. Consequently, genetic variations in TLR pathways likely dictate the intensity of gastric inflammatory reactions following *H. pylori* colonization.

Beyond inherited susceptibility, female sex emerges as a predominant non-modifiable risk determinant for autoimmune metaplastic atrophic gastritis. This sexual dimorphism reflects not merely X-chromosomal influences but also the profound immunomodulatory effects of sex hormones on target tissues and immune networks. Estrogenic compounds exert regulatory control over both innate and adaptive immunity through intricate molecular pathways. In genetically predisposed individuals, the confluence of hormonal fluctuations with environmental triggers fosters immune dysregulation, culminating in AIG and related autoimmune disorders [21].

Environmental determinants of AIG pathogenesis extend to nutritional habits and prior microbial exposures. Substantial evidence implicates pro-inflammatory dietary patterns—characterized by excessive caloric intake, refined carbohydrates, processed fats, and red meat consumption coupled with inadequate omega-3 fatty acids and dietary fiber—as potential amplifiers of autoimmune susceptibility [22]. Concurrently, viral pathogens like cytomegalovirus (CMV), herpes simplex virus, and Epstein–Barr virus have been mechanistically linked to autoimmune initiation. Recent findings corroborate the association between persistent CMV infection and autoimmune thyroid disease, while herpesvirus carriage demonstrates striking epidemiological ties to antiphospholipid syndrome in women experiencing recurrent pregnancy loss [23]. Notably, Helicobacter pylori infection occupies a central position in AIG pathogenesis. The bacterium’s molecular mimicry of gastric H^+^/K^+^-ATPase may instigate cross-reactive autoantibody production against parietal cells, thereby fueling autoimmune destruction.

Environmental factors may influence AIG onset and course through implementation of epigenetic gene regulation mechanisms as follows:

- DNA methylation of promoter regions of key immune regulatory genes can alter their expression and contribute to autoimmune processes. It has been shown that changes in methylation of genes controlling immune response and tolerance are associated with AIG and other autoimmune diseases [24,25].
- Histone modifications may affect expression of genes regulating immune tolerance and inflammatory responses. Such changes may contribute to immune dysregulation and initiation of autoimmune reactions [26].
- Small non-coding RNA molecules (miRNA) play an important role in regulating immune and inflammatory processes. Recent studies have revealed that altered expression of certain miRNAs may be associated with development of autoimmune diseases, including AIG [27,28].

The interplay between genetic predisposition and environmental factors leads to inflammation of the gastric mucosa with localization in the body and fundus of the stomach. This inflammatory process progressively results in glandular atrophy. We now examine the spectrum of histopathological alterations that may develop in the gastric mucosa:

- *H. pylori*-negative AIG (naïve).
- Combination of AIG and *H. pylori.*
- AIG in the post-eradication period of *H. pylori.*

## 2. *H. pylori*-Negative AIG (Naïve)

*H. pylori*-negative autoimmune gastritis (AIG) (naïve) develops through the interaction of genetic and environmental factors without involvement of a bacterial pathogen. This interaction leads to destruction of parietal cells by specific autoantibodies (PCAs).

The pathophysiological cascade initiated by these risk factors culminates in the targeted destruction of parietal cells by parietal cell antibodies (PCAs). These autoantibodies predominantly recognize the α- and β-subunits of the H+/K+-ATPase proton pump, an exclusive parietal cell antigen [29]. In vitro investigations have confirmed PCAs’ capacity to mediate complement-dependent cytotoxicity against gastric parietal cells [30]. Interestingly, passive transfer experiments using PCA-positive sera from AIG patients in animal models resulted in parietal cell depletion without concomitant gastric inflammation. These findings challenge the pathogenic centrality of humoral immunity in AIG development, suggesting that while PCA and intrinsic factor antibodies (IFA) serve as diagnostic serological markers, their contribution to human parietal cell apoptosis may be secondary [31]. This notion is further supported by the identification of seronegative AIG cases [32].

Emerging evidence positions autoreactive gastric mucosal T lymphocytes as the principal effectors in AIG pathogenesis [33,34]. Pioneering work by D’Elios M.M. et al. demonstrated that H+/K+-ATPase can stimulate the ex vivo proliferation of gastric CD4+ T-cell clones, predominantly of the Th1 phenotype. These activated T cells secrete proinflammatory mediators, including TNFα and interferon-gamma (IFNγ), amplifying immune responses while executing parietal cell destruction via dual mechanisms: Fas-Fas ligand (FasL) interactions and perforin–granzyme pathways. The resulting tissue transformation, perpetuated by myofibroblast activity, drives gastric body atrophy. Notably, these H+/K+-ATPase-reactive T cells additionally orchestrate B-cell activation and immunoglobulin synthesis [33].

In *H. pylori*-negative AIG (naïve), early-stage changes in the gastric body mucosa are nonspecific, featuring multifocal infiltration by lymphocytes, plasma cells, as well as mast cells and eosinophils. At this stage, manifestations of hypergastrinemia due to damage to acid-producing glands will typically include parietal cell hyperplasia. As immune inflammation progresses, diffuse lymphoplasmacytic infiltration of the lamina propria develops, with increasing severity of atrophy.

**Figure 2:**
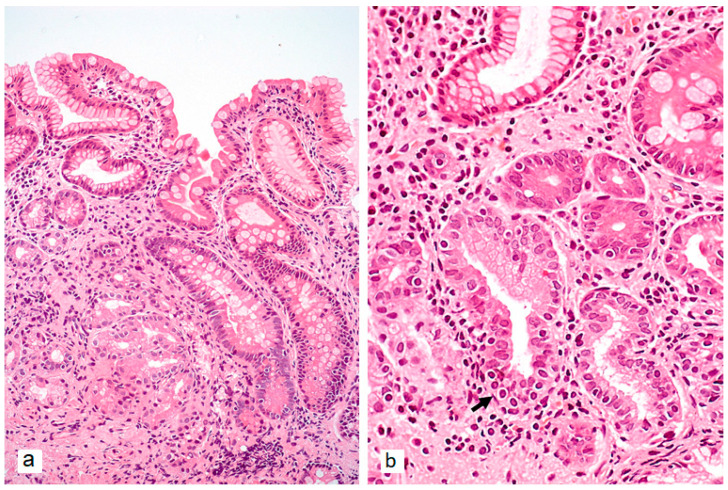
Typical morphological changes in the gastric body mucosa of a patient with autoimmune gastritis in biopsy specimens. (**a**)—Marked metaplastic atrophy: complete intestinal metaplasia in the right portion of the image, and pseudopyloric metaplasia in the left portion. (**b**)—Neuroendocrine cell hyperplasia features—linear hyperplasia: more than 5 cells with monomorphic nuclei and clear cytoplasm arranged in a row along the outer contour of glands showing pseudopyloric metaplasia (arrow). Hematoxylin and eosin staining. (**a**)—×200, (**b**)—×360.

**Figure 3:**
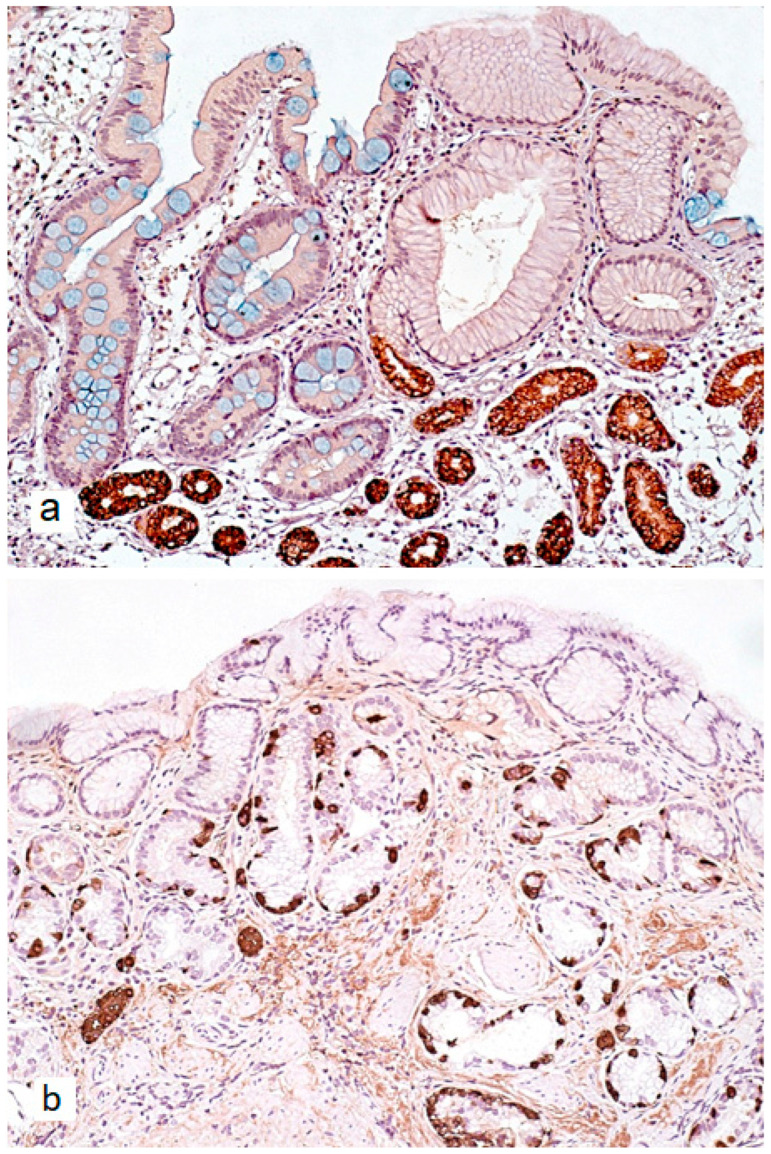
Immunohistochemical verification of autoimmune gastritis features in gastric body mucosa. (**a**)—Immunohistochemical reaction with MUC6 antibodies in areas of pseudopyloric metaplasia; absence of immunohistochemical reaction in zones of intestinal metaplasia (goblet cells stained with Alcian blue at pH = 2.5). (**b**)—Linear and micronodular (small discrete nodules) hyperplasia of neuroendocrine cells, immunohistochemical reaction with chromogranin A antibodies. (**a**)—×200, (**b**)—×160.

The characteristic progression pattern of AIG encompasses both non-atrophic and atrophic changes in the gastric mucosa. The loss of gastric body glands is accompanied by two distinct metaplastic transformations (Figure 2a and Figure 3a): pseudopyloric metaplasia (PPM) and intestinal metaplasia (IM) [35]. IM, in this context, primarily displays a complete phenotype [35]. PPM is characterized by the replacement of specialized acid-producing glands of the gastric body with cells that morphologically and functionally resemble pyloric glands. Unlike true pyloric metaplasia, PPM does not fully recapitulate normal pyloric gland architecture. Rather, it represents a phenotypic shift toward mucin-producing cells that only partially mimic the morphological and functional characteristics of true pyloric cells. Morphologically, PPM manifests through increased expression of mucin 6 (MUC6) and trefoil family factor 2 (TFF2), along with decreased expression of parietal and chief cell markers [36,37].

In 2021, Wada Y. et al. conducted a prospective study that not only documented PPM development in the gastric body as an outcome of AIG, but also proposed a “metaplastic continuum” hypothesis. Their findings suggest that, as atrophy progresses, PPM gradually leads to pyloric metaplasia [38].

PPM represents an important intermediate stage in gastric atrophy pathogenesis, as its presence is associated with an increased risk of IM and gastric epithelial dysplasia [39].

Previously, it was believed that gastric epithelial metaplasias might result from clonal expansion and reprogramming of individual progenitor cells [40]. However, contemporary studies using lineage-tracing methods have demonstrated that PPM development in AIG occurs without obligatory clonal expansion of individual progenitor cells. Instead, epithelial cells undergo plastic nonclonal reprogramming in response to chronic inflammation and immune-mediated destruction of parietal cells [41,42]. The key mechanisms underlying nonclonal epithelial plasticity in AIG likely involve changes in the local microenvironment and activation of specific signaling cascades, such as Notch, BMP (Bone Morphogenetic Protein), and Hedgehog (SHH). This microenvironment is created by the inflammatory infiltrate accompanying AIG, with production of cytokines and growth factors that facilitate plastic reprogramming of cells without selective clonal expansion [36,43].

Comprehending the polyclonal characteristics of PPM holds crucial implications for formulating preventive and therapeutic strategies against gastric precancerous states. In AIG progression, atrophic transformation may manifest with hyperplastic polypoid lesions, occasionally coinciding with pyloric gland adenoma formation, alongside invariable development of linear and micronodular enterochromaffin-like (ECL) cell hyperplasia [44].

Recent microbiome investigations by Pivetta G. et al. employing 16S rRNA sequencing revealed significant gastric microbial diversity depletion in AIG patients, with predominant colonization by *Firmicutes, Proteobacteria, Bacteroidetes, Actinobacteria, Streptococcus*, and *Prevotella* species [45]. The hypochlorhydria-induced disruption of the gastric acid barrier potentially facilitates colonization by non-*H. pylori* bacterial species, though their exact contributions to AIG-associated carcinogenesis require further elucidation.

The pathogenetic cascade in *H. pylori*-negative AIG initiates with immune-mediated targeting of acid-producing gastric mucosa (non-atrophic stage), progressing to gastric body-predominant atrophy marked by characteristic serological profiles (PCA, IFA); declining pepsinogen I/II ratios reflecting advancing atrophy; and elevated gastrin-17 levels. The resultant achlorhydria and intrinsic factor deficiency impair iron and vitamin B12 absorption, clinically manifesting as iron deficiency anemia (prevalence: 25–50%) [46,47] and pernicious anemia (incidence: 15–25%) [46,48]. These hematologic complications represent hallmark clinical presentations of advanced AIG [29].

Vitamin B12 is an essential vitamin crucial for various physiological processes such as erythrocyte development, DNA synthesis, and nervous system functioning [34]. In addition to iron and vitamin B12 deficiency, AIG patients develop deficiencies in a range of other vitamins and micronutrients, including vitamin C, vitamin D, and calcium [49,50]. The main pathogenetic mechanisms are either increased degradation or reduced absorption in the gastric mucosa, potentially due to elevated pH levels and bacterial overgrowth [51].

Special attention in AIG pathogenesis is paid to vitamin D, which has immunomodulatory effects [52]. The identification of vitamin D receptors (VDRs) in immune cells, including monocytes, dendritic cells, and activated T cells, has stimulated significant research interest in vitamin D’s immunomodulatory properties and its potential role in immune regulation [53]. VDR agonists inhibit inflammatory reactions induced by Th1 and limit subsequent Th-cell differentiation toward proinflammatory phenotype [54]. Vitamin D suppresses the proinflammatory activity of Th1 cells and secretion of proinflammatory cytokines such as interleukin 2 (IL-2), IFNγ, and TNFα [55,56,57]. The active form of vitamin D also inhibits T-cell proliferation, IL-2 expression [58], and IFNγ at both microRNA and protein levels in T cells [59]. It is assumed that insufficient vitamin D levels in the body may worsen immune regulation, potentially contributing to AIG onset or progression [51].

Furthermore, atrophy of gastric body mucosa and progressive acid secretion reduction are associated with physiological hypergastrinemia and ECL-cell hyperplasia (Figure 2b and Figure 3b), which can transform into type 1 neuroendocrine tumors (NET-1). The significantly increased risk of neuroendocrine neoplasia development confirms the need for monitoring patients with atrophic AIG [60].

The scarcity of conventional precancerous lesions (e.g., incomplete intestinal metaplasia) in AIG likely contributes to its relatively low gastric cancer risk. The predominant metaplastic phenotype—characterized by spasmolytic polypeptide expression—appears to represent an adaptive mucosal response rather than genuine neoplastic predisposition, as it lacks gastric epithelial clonal reprogramming. Nevertheless, progressive parietal cell loss and consequent hypochlorhydria induce gastric microbial dysbiosis, potentially promoting streptococcal overgrowth that may facilitate precancerous transformation and carcinogenesis [61].

The precise oncogenic potential of autoimmune gastritis continues to stimulate considerable scientific debate. Emerging evidence highlights that “naïve” AIG (H. pylori-negative, without significant inflammatory comorbidities) may demonstrate negligible gastric cancer risk. Seminal work by Rugge’s group established that such cases typically follow clinically indolent courses with minimal neoplastic progression [62,63]. However, contradictory reports persist, with multiple studies documenting elevated gastric cancer incidence among AIG patients [64,65,66]. These findings received further validation through Song et al.’s comprehensive meta-analysis demonstrating significant AIG-gastric adenocarcinoma associations [67]. Clearly, prospective investigations must elucidate additional contributory factors and molecular mechanisms underlying this complex relationship.

## 3. Combination of AIG and *H. pylori* Infection

This pathogenesis pathway of AIG involves both the mechanism of molecular mimicry between *H. pylori* antigens and host H+/K+-ATPase epitopes, and the engagement of TLR-dependent pathway inducing an immune response against *H. pylori.*

Epidemiological studies indicate a significant number of AIG patients who have either previously had or still have *H. pylori* infection, while 30% of *H. pylori*-infected individuals may test positive for PCA [68]. However, the potential role of *H. pylori* as a trigger for the immunological cascade leading to AIG development remains to be conclusively established, with current evidence requiring further validation. Amedei A. et al. demonstrated that in vivo activated CD4+ T -cells from the gastric mucosa of female AIG patients co-infected with *H. pylori* exhibit cross-reactivity to both H+/K+-ATPase and *H. pylori* antigens [69]. Based on these findings, the authors concluded that AIG pathogenesis may involve two distinct mechanisms: one is driven by a naïve autoimmune process targeting parietal cells and another that is mediated by *H. pylori* infection.

Of particular interest when studying the clinical course of AIG in *H. pylori*-infected individuals is the phenomenon of molecular mimicry between bacterial antigens and epitopes of the H+/K+-ATPase in parietal cells [70,71]. Molecular mimicry refers to a microorganism’s ability to “copy” the antigenic structure of the host, which can lead to cross-activation of the immune system and autoaggression. *H. pylori*-associated AIG represents a prime example of molecular mimicry, where activated CD4+ Th1 immune cells produced against *H. pylori* antigens cross-react with H+/K+-ATPase epitopes, triggering or exacerbating pre-existing autoimmune gastric inflammation [72].

**Figure 4:**
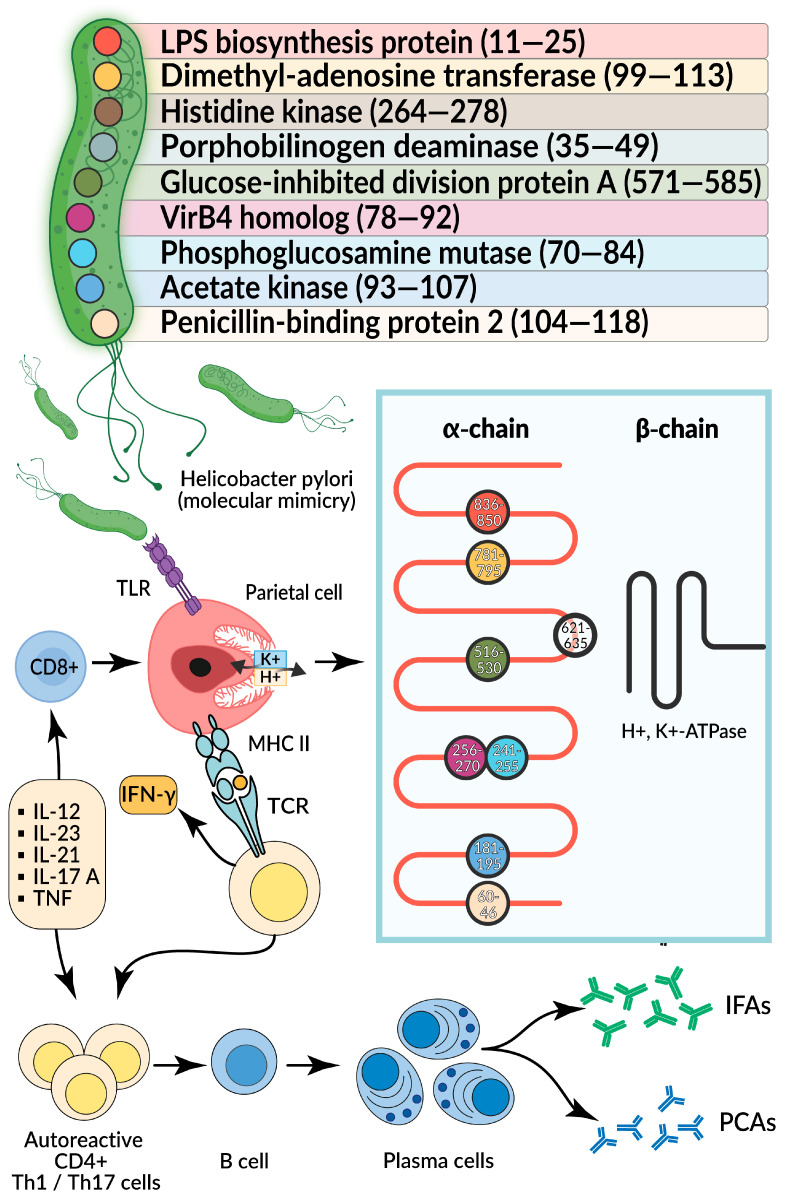
Molecular mimicry between of H. pylori and H+/K+-ATPase. Created in BioRender.com, accessed on 10 July 2025. Abbreviations: TLR—toll-like receptor, TCR—T-cell receptor, IFAs—anti-intrinsic factor antibodies, PCAs—anti-parietal cell antibodies, IFNγ—interferon γ, MHC II—major histocompatibility complex II, TNF—tumor necrosis factor, Th1—T helper cell 1, Th17—T helper cell 17, IL—interleukin.

To date, nine *H. pylori* proteins have been identified, each containing epitopes similar to those of H+/K+-ATPase [69]. As a result, parietal cells become targets for cytotoxic Th1 cells, leading to gastric body mucosa atrophy and subsequent hypochlorhydria. When H+/K+-ATPase is stimulated by a foreign pathogen, large quantities of proinflammatory cytokines are released, including IFNγ and TNFα [33]. Their local production induces MHC II molecule expression by gastric cells [73], enabling them to present H+/K+-ATPase autoantigen peptides to T cells, which differentiate into effector Th1 cells. The potential mechanism of molecular mimicry between H. pylori and H+/K+-ATPase is shown in Figure 4.

Moreover, IFNγ induces gastric epithelial cells to express co-stimulatory molecules (CD80 and CD86) and produce cathepsins involved in antigen processing. Consequently, gastric epithelial cells can function as antigen-presenting cells [74,75].

The presentation of bacterial antigens by gastric epithelial or parietal cells may activate *H. pylori*-specific FasL T cells in the stomach, which eliminate antigen-presenting cells through antigen-dependent mechanisms (perforin-mediated lysis or apoptosis induction). Additionally, the presentation of gastric H+/K+-ATPase by antigen-presenting and gastric epithelial cells to specific autoreactive T cells may stimulate further T-cell activation, leading to even a greater number of H+/K+-ATPase-sensitized Th1 cells [76,77].

From a practical standpoint, it is important to note that diagnosing *H. pylori* infection status in AIG patients is often challenging. On the one hand, false-positive results may occur due to colonization by other urease-producing bacteria. On the other hand, false negative results may arise due to a reduced number of bacterial cells or spontaneous *H. pylori* loss following severe atrophy development [78].

**Figure 5:**
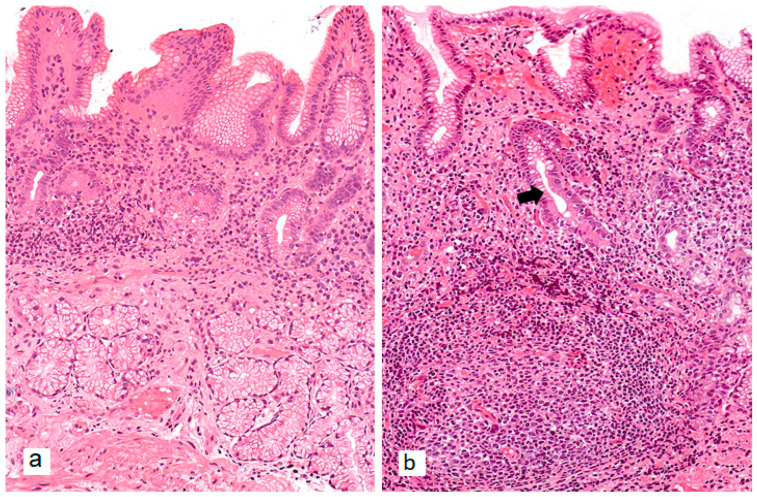
Characteristic morphological features of *H. pylori*-associated gastritis in antral gastric mucosa. (**a**)—Polymorphous inflammatory infiltrate predominantly in upper layers of lamina propria, decreased number of glands (mild absolute atrophy). (**b**)—Formation of lymphoid follicle in lamina propria, appearance of foci of metaplastic atrophy—complete intestinal metaplasia (arrow). (**a**,**b**)—×200.

In cases of combined AIG and *H. pylori* infection, histological examination reveals typical AIG changes in the gastric body with antral involvement. *H. pylori*-associated gastritis is characterized by superficial lymphoplasmacytic inflammation of the lamina propria with neutrophilic infiltration of the epithelium, while deeper mucosal layers may show lymphoid follicles (Figure 5) [79]. As *H. pylori*-associated gastritis progresses, antral atrophy foci emerge, eventually merging to form larger areas of atrophic and metaplastic mucosa that ultimately spread to the body and fundus, resulting in multifocal gastritis.

The coexistence of antral inflammation/glandular atrophy with gastric body atrophy in AIG suggests prior or current *H. pylori* infection. It has been proposed that antigenic mimicry between bacterial and parietal cell autoantigens may etiologically link AIG with prior or current *H. pylori* infection (so-called “secondary AIG”) [69].

The predictive value of OLGA/OLGIM systems for gastric epithelial neoplastic lesions in AIG requires further study. Since primary AIG does not affect the antrum, the OLGA stage in such patients never exceeds stage II. Stages III-IV strongly suggest prior *H. pylori* infection causing antral atrophy. In AIG patients with advanced OLGA stages (*H. pylori*-associated), gastric neoplasia risk ranges from 6.3% to 25% [80]. These individuals therefore require structured endoscopic surveillance.

The European Society of Gastrointestinal Endoscopy (ESGE), European Helicobacter and Microbiota Study Group (EHMSG), and European Society of Pathology (ESP) recommend that patients with extensive endoscopic changes (Kimura–Takemoto C3+ or widespread intestinal metaplasia scoring ≥5 on the endoscopic grading system EGGIM) or advanced atrophy stages morphologically assessed should undergo high-quality endoscopy every 3 years, irrespective of the individual’s country of origin [81].

## 4. AIG in the Post-Eradication Period of *H. pylori*

The third scenario essentially represents a continuation of the previous one with two potential outcomes: either resolution of gastric mucosal inflammatory changes associated with prior *H. pylori* infection, or persistence of inflammatory infiltrate (predominantly in the antrum) following pathogen eradication.

While numerous clinical studies have proven eradication therapy’s importance for cancer prevention in all patients with *H. pylori*-associated chronic gastritis, its impact on subsequent AIG course remains debatable.

Emerging evidence that H. pylori infection may trigger an autoimmune response against H+/K+-ATPase, leading to gastric body mucosal atrophy, has led researchers to hypothesize that bacterial eradication could also be effective in treating AIG [68].

The seminal 1998 case report by Stolte M. and colleagues presented a landmark observation of a 21-year-old male exhibiting histological features characteristic of active AIG, including profound lymphocytic infiltration of the lamina propria accompanied by focal gland destruction and hypertrophic residual parietal cells in the gastric body mucosa [82]. While serological evaluation failed to detect parietal cell or intrinsic factor antibodies, markedly elevated *H. pylori* IgG titers (243 U/mL) prompted eradication therapy. Remarkably, fifteen months post-treatment demonstrated not only a dramatic decline in *H. pylori* IgG to 11 U/mL, but more importantly, the complete resolution of the previously observed inflammatory infiltrates and disappearance of characteristic AIG histopathological markers. These findings led the authors to propose that *H. pylori*-associated AIG might represent a potentially curable condition through bacterial eradication, though they appropriately emphasized the necessity for confirmation through controlled prospective studies [82].

This early case established an important diagnostic precedent by confirming AIG based solely on endoscopic and histological criteria in the absence of seropositivity for conventional autoantibodies. Subsequent investigations substantially expanded these observations. Müller H. and colleagues documented that *H. pylori* eradication resulted in the resolution of active gastritis in 80% (64/80) of infected non-atrophic AIG patients during a mean follow-up period of 39.5 months [83]. Several studies further reported atrophy reversal in approximately 20% of *H. pylori*-positive patients with gastric body atrophic gastritis—a rate significantly exceeding that observed in *H. pylori*-negative cohorts [84,85].

The most recent contribution to this evolving understanding comes in 2023 from Kotera T. et al.’s report detailing the longitudinal outcomes of a 40-year-old female patient with coexisting active *H. pylori* infection and AIG following eradication therapy [86]. The authors meticulously documented progressive improvements across multiple parameters. At the seven-month follow-up, the endoscopic evaluation revealed diminished vascular pattern visibility and resolution of diffuse mucosal hyperemia, while serological assessment showed declining PCA titers (1:40), though persistent histopathological features of AIG remained evident. By twenty-six months post-eradication, further clinical improvement manifested through additional reduction in PCA titers (1:20), repopulation of parietal and chief cells in fundic glands, and normalization of serum gastrin levels to 64 pg/mL, demonstrating the potential for substantial histological recovery even in established AIG cases with appropriate intervention [86].

Mild atrophy (HR 2.14; 95% CI 1.12–4.1), moderate inflammation (HR 5.3; 95% CI 1.64–17.3), and absence of intestinal metaplasia (HR 2.4; 95% CI 1.2–4.8) were identified as histopathological predictors favoring post-eradication gastric atrophy reversal in *H. pylori*-positive AIG patients [85].

However, contradictory data exist. Sumi N. et al. demonstrated rapid post-eradication atrophy progression in an *H. pylori*-positive AIG patient [87]. In June 2023, Ihara T. et al. reported AIG onset with rapid (within 3 years) gastric body atrophy progression post-eradication in a 73-year-old woman with long-standing prior *H. pylori*-associated gastritis [88].

Despite heterogeneous data, all *H. pylori*-infected AIG patients are recommended to undergo eradication therapy with subsequent close monitoring of gastric mucosa status, considering potential case-specific interrelationships. Undoubtedly, further well-designed randomized clinical trials are needed to provide additional insights into the long-term effects of eradication therapy on AIG progression.

## 5. Conclusions

According to the classical Correa cascade model, gastric cancer development follows a defined sequence of pathological changes: from normal mucosa → non-atrophic gastritis → atrophy → intestinal metaplasia → dysplasia → adenocarcinoma. Today, *H. pylori* infection and AIG are considered leading etiological factors of gastritis with high risk of atrophy and intestinal metaplasia.

Examining molecular mechanisms of atrophy development in both *H. pylori*-negative AIG (naïve) and cases with concomitant or prior *H. pylori* infection enables more personalized gastric cancer risk assessment. Along with the risk of neuroendocrine tumors secondary to autoimmune gastric inflammation, adenocarcinoma risk increases substantially with *H. pylori* infection.

## Data Availability

Not applicable.
